# Chemotherapeutic Potential of Saikosaponin D: Experimental Evidence

**DOI:** 10.3390/jox12040027

**Published:** 2022-12-17

**Authors:** Suryaa Manoharan, Bhuvaneshwari Deivendran, Ekambaram Perumal

**Affiliations:** Molecular Toxicology Laboratory, Department of Biotechnology, Bharathiar University, Coimbatore 641 046, India

**Keywords:** apoptosis, anticancer effects, chemotherapy, *Radix bupleuri*, Saikosaponin D

## Abstract

Saikosaponin D (SSD), an active compound derived from the traditional plant *Radix bupleuri*, showcases potential in disease management owing to its antioxidant, antipyretic, and anti-inflammatory properties. The toxicological effects of SSD mainly include hepatotoxicity, neurotoxicity, hemolysis, and cardiotoxicity. SSD exhibits antitumor effects on multiple targets and has been witnessed in diverse cancer types by articulating various cell signaling pathways. As a result, carcinogenic processes such as proliferation, invasion, metastasis, and angiogenesis are inhibited, whereas apoptosis, autophagy, and differentiation are induced in several cancer cells. Since it reduces side effects and strengthens anti-cancerous benefits, SSD has been shown to have an additive or synergistic impact with chemo-preventive medicines. Regardless of its efficacy and benefits, the considerations of SSD in cancer prevention are absolutely under-researched due to its penurious bioavailability. Diverse studies have overcome the impediments of inadequate bioavailability using nanotechnology-based methods such as nanoparticle encapsulation, liposomes, and several other formulations. In this review, we emphasize the association of SSD in cancer therapeutics and the discussion of the mechanisms of action with the significance of experimental evidence.

## 1. Introduction

The phrase “chemoprevention” has established a career in the scientific community due to the increased focus on cancer prevention [1]. It has been shown that several edible plants lower the risk of cancer in humans [2]. According to epidemiological research, communities that consumed mostly “natural” (unprocessed) foods had lower cancer mortality rates [3]. Therefore, the chemo-preventive potential of several vegetable compounds was investigated. Numerous laboratory-based studies have linked this action to phytochemicals’ anticancer activities, which cause tumor cells to halt their proliferative processes. This inhibits the growth of artificially produced tumors in lab settings. The regulation of intracellular signaling pathways involved in oxidative stress, inflammation, cell proliferation, apoptosis, angiogenesis, and invasion is one way that secondary metabolites found in plants might affect cancer cells [4,5]. These include saponins, isoflavones, lycopene, curcumin, genistein, apigenin, quercetin, resveratrol, and epigallocatechin gallate [6].

In the United States, more than 1.9 million new cancer cases and 609,360 deaths are projected in 2022; lung cancer will be the leading cause of death among all cancers, accounting for around 350 daily fatalities [7]. Although cancer’s specific causes are still unknown, carcinogens are widely believed to be a key contributing factor to this dreaded illness. For the majority of malignancies, radiation and chemotherapeutic medicines such as 5-fluorouracil, cisplatin, doxorubicin, and paclitaxel are often used as treatment modalities. Chemotherapeutic medications have undesirable side effects, and are harmful to cells, even healthy ones. They affect physiological and biochemical processes and modify cell signaling pathways. However, one main focus in cancer therapy is the hunt for economical, safe, anticancer medications. Natural substances are a viable alternative to current anticancer medications to address their toxicity, side effects, and affordability. They may target a variety of important cell-signaling molecules with little negative effects. Medicinal plants and their active ingredients are beneficial in treating illnesses and preventing cancer onset, advancement, and recurrence. Additionally, natural substances or the active components of herbs play a significant supporting role in improving the effectiveness of anticancer medications and minimizing their toxicity. It has been predicted that medicine combinations made up of natural products and dietary supplements would provide the same results as traditional chemotherapy treatments with fewer side effects [8].

There are 200 species in the genus *Bupleurum* (Apiaceae), most of which are found in the north temperate zone [9]. For more than 2000 years, Asian nations, including China, South Korea, and Japan, have employed the roots of *Bupleurum* species as *Radix bupleuri*, an effective plant remedy for the treatment of digestive, endocrine, febrile, mental, oncological, and other disorders [10,11]. Triterpene saponins, known as saikosaponins (SSs), are exclusively found in *Bupleurum* plants, and present in more than 100 different forms, including Saikosaponin A, Saikosaponin B, Saikosaponin C, and Saikosaponin D [12]. 

Saponins are secondary metabolites that are part of a wide range of substances with a strong chemo-preventive potential. It has been shown that saponins have a broad range of antitumor activities. Triterpene saponins and their impact on tumor cells have drawn more attention recently. It is important to note that triterpene saponins have significant efficacy in inhibiting carcinogenesis and may function comprehensively, allowing them to interfere with tumor progression at many stages. They also display selectivity in their action on tumors and normal cells of the body. Further, marine saponins have potent inhibitory effects by impeding cell cycle arrest and death [13]. 

**Figure 1:**
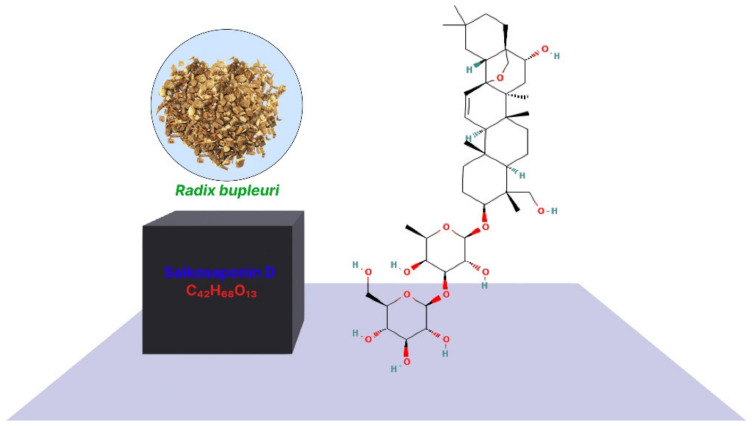
Structure of SSD and its chemical formula.

**Figure 2:**
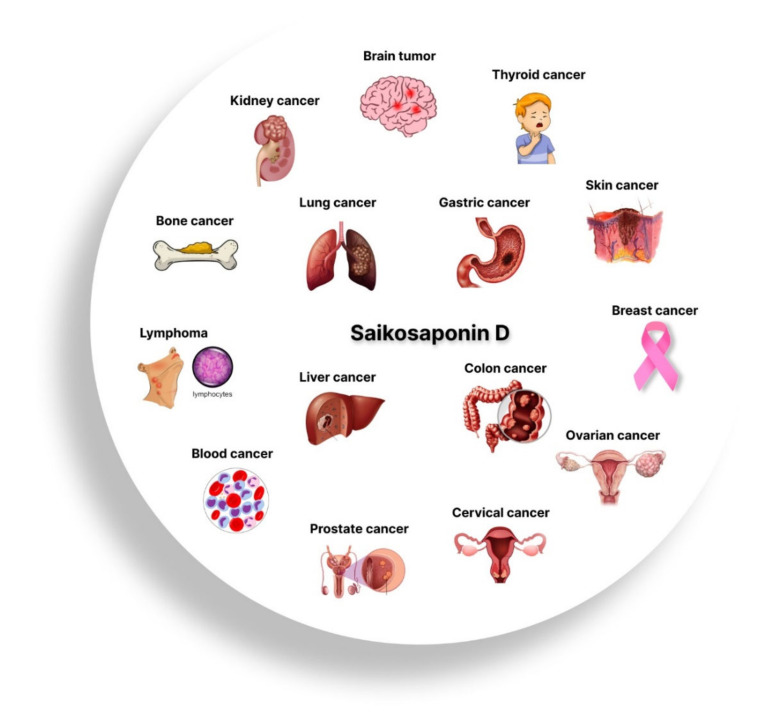
Chemotherapeutic potential of SSD in various types of cancers.

**Figure 3:**
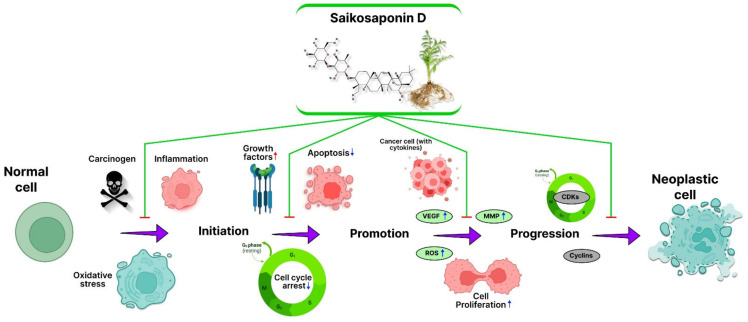
Role of SSD in cancer initiation and progression.

Additionally, *Bupleurum*’s primary secondary metabolites are SSs, which comprise approximately 7% of the dry weight of the whole root [10]. *R. bupleuri* has several pharmacological actions that are closely connected to SSs [11,14]. *R. bupleuri* is often used in clinical prescriptions to treat tumors, and according to Traditional Chinese Medicine (TCM) philosophy, it may eliminate tumor-boosting Qi and blood circulation [15,16]. As the primary bioactive elements of *R. bupleuri*, SSs have undergone substantial research to shed light on its probable antitumor effects [14]. The highest antitumor activity among SSs has been shown for SSD, whose chemical formula is C_42_H_68_O_13_, and molecular weight is 780.98 (Figure 1). SSD targets various types of cancer by multiple mechanisms (Figure 2) [17,18,19]. The major role of SSD in cancer inhibition is shown in Figure 3. Numerous triterpenoid saponins have a higher sensitivity to cancer cells than healthy cells, which raises the possibility that they might be used safely as anticarcinogens [20]. Unfortunately, data on the effectiveness and safety of SSD as a cancer preventative are dispersed, despite the possibility that SSD may be of significant importance for developing novel anticarcinogens. As a result, this study will examine the pertinent data on the pharmacokinetics, toxicity, and antitumor effects of SSD.

In order to gather information for this paper, five major databases, PubMed, Web of Science, Science Direct, Google Scholar, and ResearchGate were searched using the terms “*Radix bupleuri*,” “Bupleurum,” “Saikosaponins,” “Saikosaponin D,” and their combinations, primarily from the years 2000 to 2022. These datasets were searched between January 2022 and October 2022. As a consequence, we looked at over 150 articles, and the current work has a total of 126 references.

## 2. SSD in Inhibition of Cancer: Mechanism

### 2.1. Inflammation

A tissue’s primary response to physical, chemical, and biological stressors is inflammation. It is a defensive mechanism designed to eliminate harmful stimuli and start the healing process for the tissue [21]. However, various mediators (cytokines) produced during the inflammation and healing processes in response to noxious stimuli may have sneaky consequences and may even be detrimental [22]. In lipopolysaccharide (LPS)-exposed RAW264.7 cells, the anti-inflammatory effect of SSD and its underlying mechanism were studied. The findings showed that SSD treatment considerably reduced the levels of NO and prostaglandin E2 by inhibiting the production of inducible nitric oxide synthase (iNOS) and cyclooxygenase-2 (COX-2) levels in LPS-exposed RAW264.7 cells. Further SSD treatment significantly reduced the LPS-induced proinflammatory cytokine levels (TNF-α and IL-6) in RAW264.7 cells with SSD [23]. In another study, pretreatment with SSD prevented the LPS-induced activation of primary microglia cells in the mouse hippocampus. In vitro and in vivo overexpression of inflammatory factors, including IL-1, IL-6, and TNF-α, were decreased by SSD pretreatment, according to ELISA data. SSD administration also inhibited LPS-induced HMGB1 translocation from the nucleus to extracellular space. It lowered the levels of the TLR4, p-I-κBβ, and NF-κBp65, according to immunostaining and Western blot analysis, suggesting that this effect may have been regulated HMGB1/TLR4/NF-κB signaling [24].

### 2.2. Reactive Oxygen Species (ROS)

The imbalance between the cellular antioxidant system and free radicals is known as oxidative stress (OS). It has been demonstrated that OS-mediated damage plays a significant role in most neurodegenerative disorders [25,26]. ROS—a signaling molecule that controls various cellular processes under physiological circumstances [27,28]. SSD significantly inhibited the H_2_O_2_-induced phosphorylation of p38MAPK, JNK, and ERK in PC12 cells [29]. SSD treatment caused dose-dependent cytotoxicity in U937 and U266 cells. Apoptosis-associated proteins such as Bax, procaspase-3, and PARP were also controlled. However, a miR-657 mimic prevented the production of CHOP, p-ATF2, and PARP cleavage, which reversed the SSD-induced apoptosis [30].

### 2.3. Angiogenesis

Cancer development is significantly influenced by the tumor microenvironment, which comprises blood vessels, non-cancerous cells, and secreted substances [31]. Developing new blood arteries into tumor tissues, or tumor angiogenesis, is one of the important processes influencing the tumor microenvironment. Folkman made the first hints that angiogenesis could play a part in cancer development in 1971 [32]. In rat liver cancer tissue, SSD reduced the expression of syndecan-2, MMP-2, MMP-13, and TIMP-2 and decreased the growth of HCC [33]. SSD inhibited TNF-𝛼-induced NF-κB activation and the expression of its target genes, which are involved in cancer cell proliferation, invasion, angiogenesis, and survival, greatly potentiating TNF-𝛼-mediated cell death in HeLa and HepG2 cancer cells. Additionally, SSD demonstrated a strong ability to block TNF-𝛼-induced cancer cell invasion and angiogenesis in HUVECs while triggering death in HeLa cells by accelerating the loss of the mitochondrial membrane potential. With this discovery, they demonstrated that SSD has a significant potential to be developed as a combined adjuvant therapy for cancer patients with TNF-𝛼 [34].

### 2.4. Apoptosis

For cellular homeostasis and the elimination of aging, degenerative, dysfunctional, and damaged proteins and organelles, autophagy is crucial [35]. Numerous illnesses are linked to autophagy dysfunction, such as cancer, cardiovascular disease, and neurological disease [36]. Instead of impairing lysosome function, SSD prevented the fusion of autophagosomes with lysosomes, thus preventing autophagosome production. The genetic deletion of the autophagy-related protein five significantly decreased the amount of LC3BII accumulated by the SSD. However, it did not impact the SSD’s ability to induce apoptosis or the phosphorylation or activation of p38, activate caspase 3, or cleave PARP. According to the results of this investigation, SSD may cause apoptosis and prevent autophagy, which adds to the growing body of data linking the suppression of autophagic degradation with cell death [37]. Furthermore, the effectiveness of SSD as a cytotoxic agent was shown by apoptosis-defective or apoptosis-resistant mouse embryonic fibroblast cells that either lacked caspases 3, 7, and 8 or had the Bax-Bak double deletion. These findings provide a thorough knowledge on the mechanism of action of SSD, a new autophagic inducer with the potential to be turned into an anticancer drug that would specifically target cancer cells resistant to apoptosis [38].

### 2.5. Cell Cycle

The unchecked cell cycle is an important factor in the onset and spread of cancer. An essential tactic for preventing the spread of cancer cells and slowing the development of tumors is the coordinated activity of several biological signaling mechanisms that control apoptosis and the cell cycle [39]. Natural substances or active components from healing plants have antitumor action by inducing cell cycle arrest. According to one of these studies, SSD reduced the G2/M-phase population when there was low oxygen and increased the effects of radiation to cause G0/G1 arrest in SMMC-7721 cells. The effects of radiation to cause G0/G1 arrest, but not G2/M-phase arrest, were only amplified by SSD when exposed to normoxia [40]. Additionally, SSD slowed cell development and prevented MDA-MB-231 cells from entering the G2 phase. Cell cycle components, including cyclin A1, cyclin A2, cyclin B1, and cyclin B2, have decreased the expression of both protein and mRNA levels, which may be connected to the process of cell cycle arrest [41].

### 2.6. Signal Transducer and Activator of Transcription 3 (STAT3)

The STAT3 protein is continuously activated in tumor cells, making it an effective target for cancer prevention. By limiting the activity of STAT3, SSD is crucial in preventing cancer. As a transcription factor, STAT3 proteins direct signal transmission from the plasma membrane to the nucleus in various cellular processes [42,43]. STAT3 also regulates gene expression in response to cell-signaling proteins. A prior discovery showed that SSD mostly protected mice from APAP-induced hepatotoxicity by downregulating NF-κB and STAT3-mediated inflammatory signaling [44]. Treatment with SSD in HepG2 and SMMC-7721 cells caused the anti-apoptotic protein Bcl-2 to drop and the pro-apoptotic protein Bax to rise. SSD consistently reduced STAT3, C/EBPβ, and COX-2 mRNA expression dose-dependently [45]. SSD targeted the STAT3-SH2 domain and had a substantial STAT3-SH2 inhibitory impact on cancer [46]. While controlling cell proliferation by suppressing the p-STAT3/C/EBPβ signaling pathway that inhibits COX2 production, SSD also plays a significant role in liver cancer [47].

## 3. Anticancer Effects

The anticancer effects of SSD in various cancer types are reviewed here, and were mostly studied with biological models in vitro, as there has been limited in vivo reports. Table 1 and Table 2 provide a summary of the pertinent data.

### 3.1. Anticancer Effects of SSD In Vitro

#### 3.1.1. Lung Cancer

Radiation and chemotherapy resistance is a prevalent trait in non-small cell lung tumors, which often occur at unsuitable stages for surgery. Novel medicines are needed to mitigate the impact of the rising prevalence of lung neoplasm, since present therapeutic options are insufficient [111]. In such a situation, the antiproliferative action of SSD in A549 cells may be mediated by the p53 upregulation and Fas/FasL apoptotic pathway activation. Where the pro-apoptotic Bax protein was concerned, 20 μM SSD enhanced Bax protein levels at 12 h to 48 h [48] compared to control cells. SSD consistently decreased lung cancer cell proliferation and stopped the cell cycle in the G0/G1 phase in a dose-dependent manner. According to several earlier investigations, the overexpression of p21, p27, and p53 was primarily responsible for SSD-induced G1 phase arrest. Additionally, it caused H1299 cells to apoptosis while inhibiting STAT3 and p53 independently [49]. SSD can target the STAT3-SH2 domain and act as a major inhibitory effect against cancer, as per recent preliminary data [46]. By blocking the STAT3/Bcl-2 signaling pathway, SSD improved the anticancer impact of gefitinib in NSCLC cells, according to another in vitro study [50]. In A549 cells, SSD treatment with SP600125 resulted in reciprocal coupling of cell cycle arrest at G1/G0, G2/M, accumulated S cells, subG1 cells, subG2 cells, DNA endoreplication, and inhibited proliferation [52].

#### 3.1.2. Breast Cancer

Breast cancer is the second most prevalent illness among women, accounting for around 25% of all cancer cases [112,113]. For most patients with metastatic breast cancer, standard chemotherapy is currently the first-line or systemic therapy; however, these favored chemotherapeutic drugs are mostly linked to serious side effects and relapses after quitting the medicine [114,115]. To effectively treat patients with breast cancer, alternative medications with potential antitumor activity, reduced toxicity, and high therapeutic indices are preferred. According to research, targeted tumor treatment uses SSD-loaded biomimetic nanoparticles with T7 peptide co-coating and macrophage membrane. Nanoparticles demonstrated great cancer cell targeting selectivity and favorable immune invasion. Surprisingly, SCMNPs showed the selective accumulation of cancer cells, targeted selectivity, and improved cell endocytosis. Additionally, AKT, ERK, and VEGFR linked to the angiogenic pathway were efficiently targeted by SCMNPs to limit the development and spread of breast cancer tumors in vitro [54].

In HCC1937 cells, SSD drastically decreased the levels of the genes c-Myc, CyclinD1, and downstream targets of β-catenin [55]. In human breast cancer MDA-MB-231 cells, SSD triggered apoptosis by p38 MAPK signaling pathway activation. However, pretreatment with SB203580 decreased SSD-mediated apoptosis, PARP cleavage, and caspase3 activation in MDA-MB-231 cells. SSD administration enhanced the phosphorylation and activation of p38 MAPK [37]. SSD were effective P-gp inhibitors in MCF-7/Pac cells but not in MCF-7/Vinc cells. Doxorubicin re-accumulated after SSD therapy in MCF-7/Pac, just as it did in MCF-7/S cases. SSD is thought to be the most effective reversal agent for treating paclitaxel-resistant breast cancer [57]. SSD hindered the MDA-MB-231 cell’s ability to develop; in the low-concentration SSD group (6.25 μmol/L), and high-concentration SSD (12.50 μmol/L) groups, the proportion of cells in the G1 phase dropped considerably (*p* < 0.05). Low- and high-dosage SSD groups relative cyclin A1, A2, B1, B2 protein, and mRNA expressions considerably reduced (*p* < 0.05, *p* < 0.01) [41].

#### 3.1.3. Liver Cancer

The last stage of chronic liver disease, hepatic fibrosis, is characterized by an excessive build-up of ECM, which progresses to cirrhosis and liver cancer. Recent research has shown that SSD has a preventive effect on liver fibrosis. *R. bupleuri* is said to have a particularly powerful effect on the Liver Meridian in TCM; it is often used to treat liver problems. In the investigation of SSD’s anticancer effects, the impact of SSD on liver cancer cells has also come to the forefront. Only a tiny percentage of patients who arrive with early-stage HCC in the clinic now get successful therapy from curative procedures such as resection, liver transplantation, and ablation. However, liver cancer treatments have not yet shown to be 100 percent successful. In light of this, one of the most difficult objectives in cancer research continues to be the development of novel, efficient treatment options for liver cancer.

By inducing autophagy with the help of reducing mTOR phosphorylation, SSD promotes the radiation-induced death of SMMC-7221 and MHCC97L cells. It offers a promising strategy for the radiosensitization treatment of liver cancer [61]. SSD successfully slowed the growth of liver cancer in a dose-dependent manner. SSD at 2.5–15 μg/mL highly induced cell apoptosis by suppressing the p-STAT3/C/EBPβ signaling pathway that controls COX2 expression [47]. The detailed mechanism of SSDs and prospective targets was anticipated. The least amount of metabolite deregulation was seen in Neuropilin-1 (NRP-1) knockdown, which indicated that phospholipid metabolism and lipid transport were dramatically changed [62]. By increasing the quantity of I-κBα in the cytoplasm and decreasing the amount and activity of NF-κB in the nucleus, SSD also reduced cell survival signaling, which attenuated the BCL-xl expression in HepG2 and Hep3B cells [63].

Although metabolic-associated fatty liver disease (MAFLD) is a chronic, progressive liver disease, no efficient pharmaceutical treatments are available. In earlier studies, SSD was shown to have hepatoprotective and antisteatosis properties. By jointly regulating the PPARα activation-mediated suppression of SREBP1c-dependent Fatty acid (FA) production and stimulation of FA degradation, SSD enhanced lipid homeostasis and provided new insight into developing SSD-based treatment approaches for MAFLD [64]. Compared to the radiation-alone group, the number of autophagosomes in the SSD group was as high as 14.67 ± 0.88, which was statistically significant (*p* < 0.01). The findings demonstrated that SSD may considerably accelerate the radiation-induced autophagic development of SMMC-7721 hepatoma cells [67]. By reducing the production of the SUMO1, GLI proteins, and active sentrin/small ubiquitin-like modifier (SUMO)-specific protease 5 (SENP5), it can counteract the effects induced by hypoxia [68]. SSD interfered with p38 signaling, while SB203580, a p38-specific inhibitor, replicated SSD’s pro-apoptotic effects. Imatinib, a PDGF-R (platelet-derived growth factor-receptor) blocker, significantly decreased p38 phosphorylation while simulating SSD’s pro-apoptotic effects in LO2 cells. These findings showed that in LO2 hepatocytes, SSD disrupted the PDGF-R/p38 pathway and caused apoptosis [70].

#### 3.1.4. Kidney Cancer

The most prevalent kind of kidney cancer in adults is renal cell carcinoma (RCC), and renal transitional cell carcinoma (RTCC), mostly developed in the renal pelvis and renal parenchyma, respectively. In the USA, just one medication, sunitinib, has been licensed as adjuvant therapy for kidney cancer; in contrast, sunitinib has not received such approval in Europe. In RCC patients, maintaining renal function is crucial for lowering morbidity [116]. Increased ROS production levels are brought on by SSD therapy, along with increased IL-1, IL-6, IL-18, and TNF-α levels; decreased ROS synthesis; and inhibited NLRP3 protein expression [73]. SOD, CAT, and GPx activity were all raised during the pretreatment with SSD; however, malondialdehyde (MDA) concentration was reduced in a dose-dependent manner. At both the mRNA and protein levels, SSD dramatically boosted the expression of copper and zinc superoxide dismutase (SOD-1), CAT, GPx-1, and heat shock protein 72 (HSP72) [74]. Research on the impact of SSD in autosomal dominant polycystic kidney disease cells showed that SSD reduced proliferation by upregulating autophagy, increasing intracellular calcium levels, and activating the CaMKK-AMPK signaling cascade. These actions also blocked mTOR signaling and promoted autophagy [75]. The protein and mRNA levels of SIRT3 were dramatically increased in NRK-52E cells treated with SSD. SIRT3 downregulation eliminated SSD’s protective effects in NRK-52E cells. These results showed that SSD prevented serious glucose-induced damage to renal tubular epithelial cells by upregulating SIRT3 [76].

#### 3.1.5. Cervical Cancer

Chemo drugs, including cisplatin, carboplatin, oxaliplatin, paclitaxel, and topotecan, are used to treat cervical cancer. However, cisplatin, the primary chemotherapy treatment for individuals with cervical cancer, may become resistant to cervical cancer cells. As a result, cisplatin’s effectiveness in treating advanced or recurring cervical cancer is gravely compromised [117]. Natural bioactive substances could be a preferable option given that cisplatin can cause kidney damage (nephrotoxicity) and other frequent adverse effects such as allergy, leukopenia, neutropenia, thrombocytopenia, anemia, hepatotoxicity, and cardiotoxicity [118].

Through the build-up of ROS, SSD effectively sensitizes a variety of human cancer cells to cisplatin-induced apoptosis. The activated caspase pathway, leads to an increase in both early and late apoptotic cell death. Butylated hydroxyanisole (BHA), N-acetyl-L-cysteine (NAC), and the pan-caspase inhibitor z-VAD significantly decreased the potentiated cytotoxicity brought on by the combination of SSD and cisplatin [51]. SSD caused apoptosis and autophagic cell death by increasing autophagic flow in the cancer cells MCF-7 and HeLa. Additionally, it stimulated calcium mobilization, which activates autophagy through the CaMKK-AMPK-mTOR pathway and causes ER stress and UPR activation [38]. SSD might prevent AP-1, NF-AT, and NF-κB signaling from activating T cells. In HeLa and HepG2 cancer cells, SSD greatly increased the amount of TNF-α-mediated cell death by inhibiting TNF-α-induced NF-κB activation and the expression of its target genes, which are involved in cancer cell survival, proliferation, invasion, and angiogenesis. Additionally, SSD has shown a strong ability to stop the TNF-α-accelerated loss of mitochondrial membrane potential to cause apoptosis in HeLa cells while inducing cancer cell invasion and angiogenesis in HUVECs. SSD and TNF-α have a lot of promise for cancer patients as a combination adjuvant treatment [34].

#### 3.1.6. Blood Cancer

One of the most frequent side effects of cytotoxic anticancer drugs is cancer chemotherapy-induced neutropenia (CCIN). Even though granulocyte colony-stimulating factor (GCSF) is often utilized in clinical settings, a shortage of functionally mature neutrophils contributes to the high infection and infection-related death rates. SSD demonstrated extrinsic and intrinsic antitumor action in leukemia by targeting FTO and its downstream pathways. SSD is also considered as a possible chemotherapeutic drug for leukemia. Earlier studies reveal that SSD effectively suppressed AML leukemogenesis in a dose-dependent manner (0.5 to 1 μM). SSD restored FTO-mediated m6A hypomethylation, which lowered the stability of MTHFR and BCL-2 transcripts and proteins. MV4-11 or Kas-1-resistant cells were made susceptible to nilotinib and PKC412, while SSD was supported as a TKI-independent FTO inhibitor that can overcome resistance [79].

In HL60 cells, SSD has an inhibitory impact on cell growth and potentially increases the expression of GR mRNA. By incorporating 3H-thymidine, the antiproliferative SSD effects on HL60 cells were identified. The 3H-thymidine incorporation in HL60 cells was reduced after a 48 h treatment with 10 mg/mL SSD, and the impact was time- and dose-dependent. According to the flow cytometry examination, HL60 cells were inhibited in the G0/G1 phase [80]. SSD-induced neutrophil terminal differentiation to replenish microbicidal neutrophils. SSD also increased bactericidal activity by stimulating neutrophil differentiation in vitro (1.56, 3.12, and 6.25 μg/mL). Granulocytic differentiation brought on by SSD activated the CBLERK1/2 pathway, which aided in the therapy of CCIN [81]. Saikosaponins B3, B4, and D were discovered in the MeOH extract of the roots of *Bupleurum falcatum* L with IC_50_ values of 1.8, 3.0, and 4.3 μM. SSD blocked the interaction of selectins (E, L, and P) with THP-1 cells. Additionally, the L-selectin-mediated cell adhesion was somewhat inhibited by the aglycone structure 4 of SSD. It effectively reduced the expression of the P-selectin ligand on THP-1 cells and hindered monocyte adherence to endothelial cells. Based on these findings, we believe that SSD extracted from *B. falcatum* roots would be a promising candidate for therapeutic approaches to alleviate inflammation [82].

#### 3.1.7. Lymphoma

Lymphoma is a type of blood cancer. In the UK, it is the fifth most prevalent kind of cancer. It may have an impact on both adults and kids of any age. The effects of SSD on AP-1, NF-AT, and NF-κB signaling pathways, cytokine production, and IL-2 receptor expression in activated mouse T cells were studied. SSD prevented mouse T cell activation brought on by PMA, and Con A, in addition to suppressing the proliferative stimulation of human T cells by OKT3/CD28. SSD may thus be an option for treating autoimmune diseases caused by T cells [95]. Using a rat basophilic leukemia-2H3 cell line, SSD successfully treated allergy responses brought on by β-conglycinin. Several signaling mechanisms mediated the stimulation of rat basophilic leukemia-2H3 cells by β-conglycinin. Suppressing these crucial events in the signal transduction pathway allowed SSD to prevent rat basophilic leukemia-2H3 cell degranulation. It was hypothesized that SSD has antiallergic properties and may be used as a successful herbal treatment against soybean allergy [96].

#### 3.1.8. Brain Tumor

SSD treatment inhibited the proliferation of human malignant glioma U87 cells (1–8 μM SSD for 48 h), indicating that SSD may exert potential beneficial effects in treating malignant gliomas. SSD treatment in human glioma cells downregulated phosphorylation of Akt and ERK, upregulated JNK and caspase-3 activities, and eventually caused cell apoptosis [83]. By exercising its anti-inflammatory properties, SSD successfully alleviated LPS-induced inflammation. In particular, SSD prevented the production of pro-inflammatory cytokines and the activation of microglia caused by LPS. Additionally, the underlying process may include blocking HMGB1 translocation, releasing it into the extracellular space, and inhibiting the TLR4/NF-κB pathway [24]. SSD only showed an increase in glutamine synthetase activity, causing C6 glioma cells to differentiate into astrocytes [84]. SSD significantly suppressed the cell proliferation of DAOY cells. Additionally, it acted on SMO to block the Hh pathway, while exhibiting no effect on Hh signaling activity induced by SUFU-knockdown or GLI2 overexpression. SSD had relative selectivity for inhibition of the Hh pathway since it did not affect GLI, TCF/LEF, or NF-κB luciferase activity induced by PGE2 or TNF-α [85].

SSD (1–20 μM) inhibited PGE2 production induced by the Ca^2+^ ionophore A23187 in a concentration-dependent manner with the IC_50_ of about 3 μM. SSD possesses a dual effect: a reduction in PGE2 synthesis brought on by A23187 without a direct reduction in cyclooxygenase activity; and an elevation of [Ca^2+^] ions that are attributed to Ca^2+^ release from intracellular stores [86]. Hoechst 33342/PI, annexin/PI, and TUNEL staining tests were used to validate the neuroprotective effects of SSD. The differential modulation of mitochondrial and nuclear GR translocation, partial correction of mitochondrial dysfunction, blockage of the mitochondrial apoptotic pathway, and selective activation of the GR-dependent survival pathway were a few ways that SSD showed its antiapoptotic effects [87]. The ROS inhibitor prevented apoptosis brought on by MAPK activation and cellular oxidative damage, and SSD dramatically decreased H_2_O_2_-induced ROS build-up. This work demonstrated that SSD inhibits MAPK-dependent oxidative damage and H_2_O_2_-induced PC12 cell death by eliminating ROS (200, 300, and 400 μg/mL SSD for 24 h). This suggests that SSD may act as a possible antioxidant with therapeutic benefits for neurological oxidative disorders [29].

#### 3.1.9. Ovarian Cancer

A malignant growth called ovarian cancer develops in the tissues of the ovary. Age raises the risk of ovarian cancer. Both women and those born with a feminine gender preference may have ovarian cancer. About 1 in 78 people may acquire ovarian cancer in their lifetime. The naturally occurring substances called SSs, obtained from *R. bupleuri*, have been shown to exhibit anticancer action in a number of cancer cell lines. First-line anticancer medications for ovarian cancer include cisplatin and its variants (OVCA). The sensitivity of cancer cells might be significantly increased by combining SSD with cisplatin. SSs alter the redox state of cancer cells, making them more susceptible to cell death caused by cisplatin. The addition of SSD may significantly increase the susceptibility of cancer cells to cisplatin. Because of the cotreated cell’s characteristic apoptotic shape, it increased early apoptotic, late apoptotic cell population, and caspase activation. The increased cell deaths in SSs and cisplatin-cotreated cells were mostly apoptotic. The pan-caspase inhibitor zVAD-FMK may effectively suppress the chemosensitization action of SSD. When cancer cells were pretreated with ROS scavengers before SSD exposure, the potentiated cytotoxicity was successfully suppressed [51]. Regardless of their p53 status, chemoresistant OVCA cells were made more susceptible to cisplatin by SSD via the development of mitochondrial fragmentation and G2/M arrest. Ca^2+^ signaling, upregulation of the mitochondrial fission proteins Dynamin-related protein 1 (Drp1) and optic atrophy 1 (Opa1), and reduction in mitochondrial membrane potential (MMP) were the mechanisms by which SSD was mediated. In the presence of cisplatin, SSD inhibited PPM1D and boosted the phosphorylation of Cdc25c, Cdk1, and Chk1. To treat chemoresistant OVCA, SSD may be considered a new adjuvant [88].

#### 3.1.10. Prostate Cancer

A kind of cancer that appears in the prostate gland is called prostate cancer. It ranks as the fifth-leading factor in men’s cancer-related deaths. Due to its ability to stop the multiplication of many cancer cell lines, SSD has drawn a lot of interest. The effect of SSD on the DU145 human prostate cancer cell line induces apoptosis. Treatment with SSD caused the DU145 cells to increase in a concentration-dependent manner and be inhibited. Maximum inhibition was reached at 24 h with 50 μM SSD, which prevented 80% of the growth of DU145 cells and had an IC_50_-value of 10 μM. Through the overexpression of p53 and p21, SSD stopped the cell cycle in the G0/G1 phase. SSD used the intrinsic apoptotic pathway to cause apoptosis in DU145 cells. SSD may emerge as a top candidate medication for the treatment of prostate cancer [89]. SSD exhibits antimetastatic effects on PC through the reduction of EMT and MMP2/9 expression linked to cell migration and cell invasion, as well as CSCs. These results might be the outcome of SSD therapy inhibiting GSK3β/β-catenin signaling in CWR22Rv1 cells. Targeting EMT and CSC by SSD’s anticancer action suggests that SSD may be a powerful drug for CRPC treatment [90].

#### 3.1.11. Bone Cancer

Although bone cancer may start in any bone in the body, the pelvis or the long bones in the arms and legs are often affected. Less than 1% of all malignancies are bone cancers, making them very uncommon. Benign bone tumors are significantly more prevalent than malignant ones. Despite improvements in bone cancer detection and treatment, including surgery, chemotherapy, and MRI imaging, the overall survival rate is still low because of the disease’s invasiveness and distant metastases. Therefore, the therapy needs new, efficient, and trustworthy procedures. The potential of SSD as an antitumor drug in bone cancer makes an effective treatment. The administration of SSD at 80 μmol/L dramatically reduced the proliferation of MG-63 and 143B. In contrast to cyclinD1, SSD treatment upregulated tumor protein p53 and its downstream targets, including p21, p27, B-cell lymphoma 2 like protein 4, and cleaved caspase 3. It has been hypothesized that SSD was a functional tumor suppressor that prevented osteosarcoma growth by activating the p53 signaling pathway, and it could one day be employed to treat osteosarcoma [91]. Across both the death receptor route and the mitochondrial apoptotic pathway, SSD can cause apoptosis whether used alone or in combination with SP600125 (24 h of treatment with 20 μM SSD and 20 μM SP600125). SSD alone and in conjunction with SP600125 are both potent options for the chemoprevention and therapy of osteosarcoma because they have a synergistic antitumor impact on U-2OS cells [92].

#### 3.1.12. Colon Cancer

One of the most prevalent malignant tumors of the digestive system in people is colon cancer. Polyps (growths) in the inner lining of the colon cause colon cancer. With around 10% of all occurrences worldwide, colorectal cancer is the third most prevalent kind of cancer. In Chinese herbal medicine, *Bupleurum* is a significant and often used for the treatment of tumors in the digestive system. According to Chinese Pharmacopoeia’s quality control of the herb, SSD has an antitumor effect on colon cancer. SSD may control the PI3K-Akt-mTOR pathway to prevent colon cancer. Human colon cancer SW480 and SW60 cells showed the dose-dependent induction of apoptosis when SSD triggered the Bax/Bcl2 and caspase-9/caspase-3 cascades. SSD at a dosage of 50 μg/mL for 24 h may successfully trigger the apoptosis of SW480 and SW620 cells. According to its clinical effectiveness, SSD is anticipated to be a successful therapy for colon cancer [93]. At a dosage of 10 mg/L of SSD for 24 h, SSD caused the apoptosis of HT29 cells through the death receptor pathway (TRAIL, TRAIL-R, caspase10, and caspase8). Fourteen different apoptotic gene expression levels dramatically altered in cells treated with SSD. SSD could thus provide promises for colon cancer therapy [94].

#### 3.1.13. Thyroid Cancer

Thyroid cancer occurs when cells reproduce rapidly. It is a type of tumor that is generally seen as a mass, nodule, or thyroid gland located at the throat base. Because of the anticancer effects of SSD, it is defined as the new potential drug candidate for thyroid cancer. Three human anaplastic thyroid cancer cell lines, 8305C, ARO, and SW1736, showed decreased cell growth after SSD therapy. SSD caused G1-phase cell cycle arrest and increased cell death (10 μmol/L, 15 μmol/L, and 20 μmol/L for 24 h). The effects of SSD therapy included increased p53, Bax, and decreased Bcl-2 expressions. The administration of SSD resulted in a notable upregulation of p21 and a downregulation of CDK2 and cyclin D1. For human undifferentiated thyroid cancer, SSD may be a novel, effective chemo-preventive medication for human undifferentiated thyroid cancer [97].

#### 3.1.14. Skin Cancer

Skin cancer is the uncontrolled growth of epidermic cells, which causes mutations by DNA damage. These mutations cause rapid multiplication of epidermic cells and form malignant tumors. SSD nanoparticles exhibit enhanced antimelanoma action and induce death in melanoma cells through the mitochondrial pathway, mediated by increased cytochrome c levels, and activation of caspase 9, JNK, p38, and p53 signaling. SSD nanoparticles are now more soluble and might one day be employed to treat melanoma [98].

#### 3.1.15. Stomach Cancer

Stomach cancer is a type of tumor which occurs in the stomach internal lining and progress deeply in the barriers. These tumors expand to the close contact cells of the liver and pancreas. SSD is considered as the potential ingredient in TCM and also shows substantial growth inhibition and activation of apoptosis in a variety of human solid cancers. By focusing on the MKK4-JNK signaling pathway, SSD was able to inhibit tumor development and encourage death in pancreatic cancer cells, suggesting the potential for further therapeutic options against stomach cancer. SSD strongly inhibited BxPC3, Pan02, and PANC1 cells in a concentration- and time-dependent manner. When the SSD concentration was raised to 4 μM, the inhibitory rate decreased to 31.61 percent. SSD may become a top candidate medicine for treating pancreatic cancer [99]. SSD increased the GC cells’ sensitivity to DDP, reducing GC cell migration and proliferation, causing apoptosis, and promoting autophagy. Particularly in SGC-7901/DDP cells, SSD enhanced the impact of the DDP-induced elevation of cleaved caspase 3 levels and the inhibition of the NF-κB pathway. This suggests that SSD could help in treating GC patients who have developed DDP resistance [100].

### 3.2. Anticancer Effects of SSD In Vivo

#### 3.2.1. Lung Cancer

Gefitinib’s antitumor activity was improved by SSD, according to animal tests. It showed that gefitinib and SSD had a stronger anticancer impact on NSCLC cells, and that STAT3/Bcl-2 signaling pathway suppression was a molecular mechanism behind this mechanism, putting out a viable strategy for treating NSCLC patients who are EGFR-TKI resistant [50]. In mice exposed to BLM, lung alveolitis, pulmonary fibrosis, and cell apoptosis were all reduced by SSD. Additionally, in both 14 and 28 days, SSD decreased the expression of caspase-3, FN, Wnt, and β-catenin. This suggests the possibility that the medication for SSD might be an effective treatment option for PF [53].

#### 3.2.2. Breast Cancer

The research focused on creating macrophage membranes, and T7 peptide-coated biomimetic nanoparticles with SSD loaded into them for targeted tumor treatment via modification of the angiogenic signaling pathway. Nanoparticles showed good immune evasion and great selectivity for targeting cancer cells. SCMNPs cannot only remove the local tumor but also inhibit the spread of the tumor by producing a strong abscopal antitumor impact. Mechanistically, SCMNP therapy significantly reduced the levels of p-Akt and p-ERK proteins in the mice tumor, indicating that the drug’s substantial suppressive effects on tumor development in vivo may be caused by angiogenic malfunction [54]. By preventing the expression of P-gp, SSD and Dox showed a clear tumor-suppressive impact in a nude mice xenograft model, indicating that using SSD in conjunction with other treatments might be a fruitful way to treat P-gp-mediated MDR. SSD may significantly inhibit the MCF-7/ADR cell xenograft’s growth. According to this investigation, SSD might overcome MDR in vivo without causing other harmful side effects [58].

#### 3.2.3. Liver Cancer

SSD slows tumor development and makes HCC cells more susceptible to HSVtk/GCV. Following an HSVtk/Hep3B cell inoculation, SSD, GCV, or a combination of both were administered to BALB/c nude mice [68]. SSD controlled lipid metabolism by reducing the mRNA levels of adipogenic genes and increasing those of genes involved in FA oxidation. SSD successfully treated HFSW-induced fatty liver by adjusting the balance between lipid storage and energy consumption. This is solid justification for enhancing novel SSD-based medication candidates for managing MAFLD as a main or adjuvant therapy [64]. By increasing p53 expression as a result of inhibiting HIF-1α expression, the radiosensitization impact of SSD in hypoxic circumstances may be shown. Some evidence suggests SSD may be a radiation sensitizer for liver cancer [102]. Furthermore, 200 mg/kg and 400 mg/kg of SSD are the most efficacious dosages for determining the molecular mechanisms against D-galactosamine/lipopolysaccharide-induced hepatotoxicity. On the hepatotoxicity brought on by D-galactosamine/lipopolysaccharide, SSD exerts protective effects [24].

Using a rat model of DEN-induced HCC, SSD inhibited C/EBPβ and COX-2 to prevent the development of hepatocarcinogenesis. This work offers valid explanations of the mechanism of HCC and crucial experimental data for the future therapeutic use of SSD [119]. SSD mostly downregulated NF-κB and STAT3-mediated inflammatory pathways to protect mice against APAP-induced hepatotoxicity. This research (SSD 2 mg/kg twice daily for five days) [44] demonstrates one of the probable mechanisms of hepatoprotection brought on by SSD. Because SSD increased I-κBα activity in the liver and decreased liver TNF-α, IL-6, and NF-κBp65 expression, it may have hepatoprotective and anti-inflammatory actions that reduce CCl4-induced hepatic fibrosis in rats [104]. By preventing the growth of HCC and reducing the expression of syndecan-2, MMP-2, MMP-13, and TIMP-2 in rat HCC liver tissue, SSD was also utilized to treat chronic liver disease. In HCC, SSD may suppress carcinogenesis by lowering cell–ECM contact and slowing angiogenesis and ECM remodeling. SSD’s potential ability to suppress MMP-2 and MMP-13 activity may help to prevent HCC invasion and metastasis [33]. SSD (1.0 mg/kg once a day for 18 weeks) may prevent the angiogenesis of DEN-induced hepatocarcinogenesis, and the mechanism may be connected to the downregulation of Ang-2 and VEGF expressions [106].

#### 3.2.4. Kidney Cancer

In C57BL/6J mice, SSD avoided AKI development and decreased inflammation, while the ROS inhibitor improved SSD anti-inflammatory activities. Through the NLRP3 inflammasome and SIRT1, SSD decreased inflammation in mice. SSD (10 mg/kg, i.p.) was administered to SSD group mice for three days. To gain an understanding of the activation of AKI and its therapeutic use, SSD may be useful [73]. By lowering TGF-β1 expression and decreasing the invasion of macrophages and CD8^+^ T cells, SSD (0.6 or 1.8 mg/kg of SSD in Wistar rats) slowed the course of mesangial proliferative glomerulonephritis [107]. One week of intragastric injection of SSD to C57BL/6JNifdc mice lowered the renal inflammation and cell death brought on by sepsis. SSD inhibits MMP9 expression and downregulates TCF7 to reduce FOSL1 transcription [108].

#### 3.2.5. Blood Cancer

In mice using the CCIN model, SSD-induced neutrophils could combat infection without significantly increasing leukocyte numbers. In the CCIN mouse model, SSD increased bactericidal activity by promoting neutrophil differentiation. By stimulating the CBL-ERK1/2 pathway, SSD might cause granulocytic differentiation to produce functioning, mature neutrophils that can fight infection. SSD could provide a new method for treating CCIN [81].

#### 3.2.6. Brain Tumor

The SSD dose with the greatest tumor-inhibitory impact was 1.0 mg/kg (40.96%). SSD paired with the oxaliplatin group led to greater induction of apoptosis in nude mice harboring A549 cells. Inducing apoptosis of A549 cells in nude mice with the help of SSD and oxaliplatin may be accomplished via downregulating the expression of COX-2 [109]. In MB allografts, SSD (10 mg/kg, IP) demonstrated good in vivo inhibitory action, with tumor growth inhibition ratios of around 50% and 70%, respectively. By blocking the Hedgehog pathway and concentrating on SMO, it has been proposed that SSD dramatically reduced tumor development in MB models [85]. Male ICR mice pretreated with SSD (1 mg/kg) for 7 days inhibited the LPS-induced activation of microglial cells. They decreased the microglia’s morphological changes, which included soma expansion, the distal process shortening, and phagocytic amebiform cells [24]. By lowering amyloid plaque deposition and glial cell activation in the hippocampus, SSD may alleviate memory deficits in 3xTg mice. SSD-mediated protection against neuroinflammation and apoptosis may be linked to inhibiting the NF-κB signaling pathway (SSD treatment for 28 days). In the future, it was hypothesized that SSD might be used as a therapeutic agent in the treatment of AD [110].

#### 3.2.7. Thyroid Cancer

SSD decreased the proliferation of anaplastic thyroid tumor cells. SSD significantly decreased thyroid tumor size and mass. SSD may be a novel, very effective chemo-preventive medication option for treating human undifferentiated thyroid cancer, according to this report [97].

## 4. Conclusions and Future Perspectives

Cancer remains the principal cause of mortality regardless of major recent advancements in therapeutics. Anticancer medications successfully treat cancer but can have negative side effects, such as physiological and biochemical changes, exhaustion, alopecia, infection, nausea, and vomiting. Through the modulation of numerous biological functions, natural products are demonstrated to have a key responsibility in preventing and suppressing cancer. SSD exhibits an anti-cancerous action by suppressing the initiation, promotion, and advancement phases. It has been shown that combining SSD with chemo-preventive drugs enhances their anti-cancerous effectiveness and lessens their toxicities.

**Figure 4:**
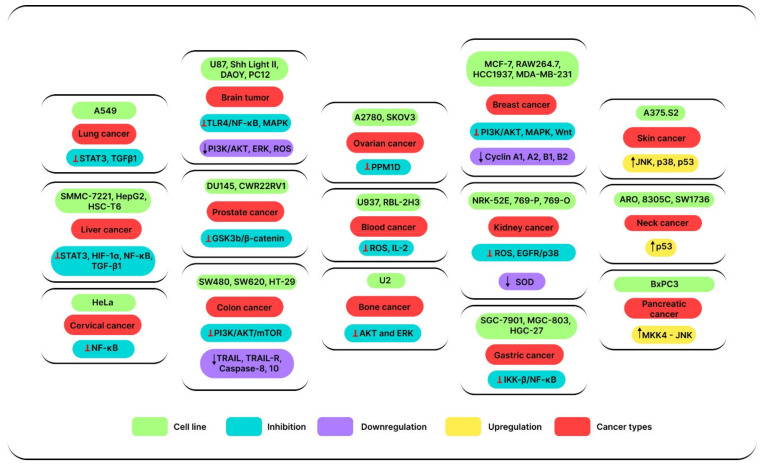
Chemotherapeutic potential of SSD in vitro.

Due to the ubiquitous hostile responses of conventional anticancer strategies and the notable drug resistance that harms the effectiveness of anticarcinogens, the creation of novel anticarcinogens has been a focus of pharmaceutical anticancer research [120,121,122]. Prolonged drug usage in humans is the foundation of traditional medical experience. We can examine the mechanisms underlying these conventional therapeutic experiences at a deeper level thanks to the growing body of molecular research, our extensive apprehension of the tumor genesis and development together with the drug mechanisms, and our discoveries and innovations in the field of drug development [119,123]. SSD has received a lot of attention and has been shown to have an antitumor effect on various tumors, since it is the most antitumor ingredient in *R. bupleuri*. The antitumor effects of SSD, on the other hand, have multiple targets, such as the inhibition of cell proliferation, cell invasion, metastasis, and angiogenesis, as well as the induction of cell apoptosis, autophagy, and differentiation, thereby challenging the tumor cells that are susceptible to establish drug resistance to specific-targeted drugs. According to study data, SSD showed greater antitumor efficacy in vitro (Figure 4) than certain well-known antitumor medications, including taxol and SP600125. Due to SSD’s undeniable antitumor properties, researchers recognized it as a viable natural compound for the treatment of various tumors. However, the antitumor effects and SSD mechanisms in vivo still need additional study and corroboration. Current findings, on the other hand, mostly concentrate on in vitro investigations.

A significant challenge in current cancer therapeutics is tumor drug resistance [124]. The increased sensitivity of cancer cells to various chemotherapeutic agents has demonstrated that SSD works well in synergy with some of them and reduces drug resistance. SSD abolished drug resistance at doses below 0.6 μM, while having no adverse side effects. As a result, it is possible to create a safe chemotherapeutic sensitizer using SSD at lower dosages. As per the earlier reports, SSD suppresses proliferation and increases apoptosis in renal cell carcinoma cells by blocking the p38 pathway. On the other hand, SSD activates the p38 pathway to promote apoptosis in breast cancer cells. Distinct tumor cells respond differently to p38 activation, sometimes even in entirely contradictory ways, depending on the discreetness of various isomers to substrates and the distribution of peculiar tissues. According to Martnez-Limón et al. [125], the control mechanisms of p38 in the proliferation, differentiation, metastasis, and apoptosis of many tumors are still very intricate to fully comprehend. Therefore, utilizing more focused antibodies of the appropriate isoform of p38 may be useful in investigating the anticancer mechanisms of SSD in various tumors.

SSD was found to have a structural similarity to estrogen, and to demonstrate action analogous to estrogen by stimulating the ERα-mediated pathway, supplementary to exercising the proliferative induction on MCF-7 cells, regardless of the evidence illustrating that SSD played an antitumor role in the reproductive system of females [126]. These data indicate that additional research on the effects of SSD in these tumors is required, as well as consideration of the possible health implications of SSD in patients with ERα-positive malignancies. Despite being a member of the group of triterpenoid saponins with weak water solubility, SSD may have a better bioavailability than we had anticipated. Simultaneously, research on SSD metabolism has shown that SSD can be converted into specific metabolites due to certain transformation factors in vivo. Although numerous in vitro studies have been carried out to date and showcased the immense potential of SSD and their synergistic actions with other bioactive compounds to inhibit diverse cancer types, their prospects in in vivo are under-studied, and further research has to be carried out to exploit their potential to the fullest.

SSD offers a viable anticancer drug in light of the scientific research that is currently accessible and covered above. However, most SSD research is constrained by the study design, experimental bias, variable outcomes, and lack of repeatability among study cohorts. These restrictions start with SSD research conducted in vitro at concentrations that are not feasible under physiological circumstances in vivo. The choice of the best incubation durations and delivery sequences for the cotreatment tests, in which SSD is evaluated with other active compounds such as chemotherapeutics, might also impact the research reproducibility. To translate SSD in clinical trials for safety and efficacy, high-quality experimental research designs are necessarily based on the experimental constraints outlined above. To create innovative combination treatments using SSD, a deeper comprehension and identification of the pharmacological targets interacting with SSD in signaling cascades governing tumor initiation and progression are also required. For unique and cutting-edge insights to create targeted SSD therapies in vivo, more experimental research designs examining the interaction of protein targets with SSD are required.

## Figures and Tables

**Table 1:** Anticancer effects of SSD in vitro.

S. No.	Cancer Type	Cell Line	Dose/Conc.	Exposure (Hours)	Effects on Signaling Pathways	Reference
1.	Lung cancer	A549	1–20 μM	24 h	♦ Fas/FasL apoptotic system and the p53 pathway, ◙ G1-phase ◘, proliferation restraint.	[48]
2.	Lung cancer	A549, H1299	5–20 μM	24 h	◙ STAT3 pathway, ◘ G0/G1-phase arrest, ◙ proliferation, ◘ apoptosis.	[49]
3.	Lung cancer	HCC827, H1975, PC-9, HCC827/GR	5–40 μM	24 h	◙ STAT3 pathway, ◙ proliferation, ◘ apoptosis, Chemosensitization (gefitinib).	[50]
4.	Lung cancer	A549	2 μM	48 h	◘ ROS accumulation, Enhancement of apoptosis, Chemosensitization (CDDP).	[51]
5.	Lung cancer	A549	0.5, 2 μM	12–48 h	◙ Proliferation and migration by diminishing the JNK/pJNK negatively regulating p53, ◙ G1 and G2 cell cycle.	[52]
6.	Lung cancer	HELF	2.5, 5, 10 μg/mL	96 h	◙ Proliferation and TGF-β1 expression, ◙ epithelium-mesenchymal transition and alveolar epithelial cells.	[53]
7.	Breast cancer	MCF-7, 4T-1, RAW 264.7 LO-2	0–60 μM	24 or 48 h	◘ Cell death, ◙ PI3K/Akt and MAPK/ERK pathways.	[54]
8.	Breast cancer	HCC1937	13–100 μM	2–24 h	◙ Wnt/β-catenin pathway, ◙ proliferation, ◘ apoptosis.	[55]
9.	Breast cancer	SK-BR-3, MCF -7, HBL-100	2.5, 5, and 10 mM	48 h.	Bcl - 2, proto-oncogenetyrosine-protein kinase src are regulated, ◙ cell proliferation by estrogen receptors.	[56]
10.	Breast cancer	MDA-MB-231	6–15 μM	24 h	♦ p38 pathway, ◙ viability, ◘ apoptosis.	[37]
11.	Breast cancer	MCF-7	40 μg/mL	72 h	◙ P-gp in MCF-7/Pac, amplify the antiproliferative impact, MDR reversal in MCF-7 sublines that are resistant.	[57]
12.	Breast cancer	MCF-7	10 μM	4 h	◙ SERCA, ♦ CaMKKβ-AMPK-mTOR signaling cascade, ER stress, and UPR, ◘ apoptosis and autophagy.	[38]
13.	Breast cancer	MCF-7/ADR	0.13–0.6 μM	48 h	◙ P-gp expression, MDR reversal without harmful consequences, Chemosensitization (doxorubicin).	[58]
14.	Breast cancer	MCF-7/ADR, MCF-7	0.13–0.6 μM	48 h	▼MDR1/P-gp, Reversal of MDR without toxic effect, Chemosensitization (ADR).	[59]
15.	Breast cancer	MDA-MB-231	6.25 μmol/L–12.50 μmol/L	12, 24, 48 h	◙ MDA-MB-231 cells in G2 phase, cell cycle arrest, ▼cyclin A1, cyclin A2, cyclin B1, and cyclin B2.	[41]
16.	Liver cancer	SMMC-7721	3.2–19.2 μM	24 h, 48 h, 72 h	◙ p-STAT3/HIF-1α pathway and ◘ Suppression of COX-2 expression, ◙ proliferation.	[60]
17.	Liver cancer	SMMC-7721, MHCC97L	3 μg/mL	2 h	▲ Radiation, ◘ apoptosis by promoting autophagy via ◙ mTOR phosphorylation	[61]
18.	Liver cancer	SMMC-7721, HepG2	3.2–19.2 μM	24 h, 48 h, 72 h	◙ p-STAT3/C/EBPβ pathway and COX-2 expression, ◙ proliferation, ◘apoptosis.	[47]
19.	Liver cancer	HepG2	1.75, 3.75, 7.5, 15 μM	24 h	NRP -1 knockdown dramatically changed the lipid transport, phospholipid metabolism, and enhanced anti-hepatoma action.	[62]
20.	Liver cancer	HepG2, Hep3B	1–10 μM	12 h–48 h	♦ p53 and Fas/FasL pathway, ◙ NF-κB pathway, ◘ G1- cell cycle arrest, ◙ proliferation, ◘ apoptosis.	[63]
21.	Liver cancer	HepG2	2.5, 5 and 10 μM	16 h	◙ Hepatic FA biosynthesis, PPAR activation modifies the INSIGs/SREBP1c pathway.	[64]
22.	Liver cancer	HepG2	10 μM	24 h	◙ NF-κB ♦, ◙ proliferation, angiogenesis, and invasion, ◘ apoptosis.	[34]
23.	Liver cancer	Hepatocyte	100, 200, and 400 ng/mL	24 h	Enhance cell viability, ◙ apoptosis, ◙ mortality of hepatocytes.	[65]
24.	Liver cancer	SMMC-7721 HepG2	1.28, 3.84 μM 3 μg/ml	2 h	♦ p53 pathway, ▲ G0/G1 arrest, ◘ G2/M-phase arrest under hypoxia, ◘ apoptosis, Radiosensitization. Bax▲, HIF-1α▼.	[40]
25.	Liver cancer	Hep3B	5 μM	24 h	◘ Cell apoptosis via both mechanisms reliant on and independent of caspase-3.	[66]
26.	Liver cancer	SMMC-7721, MHCC97L	3.84 μM	24 h, 48 h, and 72 h	Suppression of mTOR pathway, ◙ proliferation, ◘ autophagy formation, radiosensitization.	[67]
27.	Liver cancer	Hep3B	2–15 μM	48 h	▲SENP5 expression and subsequent ◙ Gli1 SUMOylation, ◙ SHh pathway, ◙ viability, invasion and migration, ◘ apoptosis, chemosensitization (HSVtk/GCV).	[68]
28.	Liver cancer	HepG2	5 μg/mL–20 μg/mL	6 h	◘ Apoptosis, ♦ caspases-3 and caspases-7.	[69]
29.	Liver cancer	HSC-T6 LO2	1 μM 0.4 μM	24 h	▼ Expression of a smooth muscle actin, TGF-β1, ERK1/2, PDGFR, TGF-β1R, extracellular matrix regulated kinase 1, and connective tissue growth factor.	[70]
30.	Liver cancer	SMMC-7721	5.0, 7.5, 10.0, 12.5, 15.0, and 17.5 mg/L	24 h, 48 h, and 72 h	◘ Autophagy through ▲ expression of BECN1, ◙ SMMC-7721 proliferation.	[71]
31.	Liver cancer	Hep3B	-	-	Hep3B cells may be made to undergo apoptosis by using caspase-3-independent mechanisms.	[72]
32.	Kidney cancer	Mouse renal tubular epithelial (mTE) cells	-	-	Prevents AKI via ◙ NLRP3 inflammasome by SIRT1, ROS ◙	[73]
33.	Kidney cancer	LLC-PK1	1 or 3 μg/mL	24 h	▲ Expression of anti-oxidant enzymes (SOD, CAT, GPx) and HSP72.	[74]
34.	Kidney cancer	UCL93 and OX161	5.0 μM	24 h	CaMKK-AMPK-mTOR signaling pathway to ◘ autophagy, ◙ SERCA to increase calcium levels.	[75]
35.	Kidney cancer	NRK-52E	45 and 60 μM	24 h or 48 h	Attenuates oxidative injury via ▲ of SirT3, SOD activity▼ and SIRT3 expression ▲.	[76]
36.	Kidney cancer	HK-2	20–150 μM	0–48 h	ROS-mediated ♦ of MAPK and NF-κB signal pathways.	[77]
37.	Kidney cancer	769-P, 786-O	10–20 μM	48 h	◙ EGFR/p38 pathway, ▲ p53. ◘ apoptosis, ◘ G0/G1-phase arrest, ◙ proliferation.	[78]
38.	Cervical cancer	HeLa, Siha	2 μM	24 h, 36 h	◘ Intracellular ROS accumulation, Enhancement of apoptosis, Chemosensitization (CDDP).	[51]
39.	Cervical cancer	HeLa	10 μM	24 h	◙ SERCA, ♦ CaMKK-AMPK-mTOR kinase signaling cascade, ER stress and UPR, ◘ apoptosis and autophagy.	[38]
40.	Cervical cancer	HeLa	10 μM	0–24 h	◙ NF-κB pathway, ◙ proliferation, angiogenesis and invasion. ◘ apoptosis, Chemosensitization (TNF-α).	[34]
41.	Blood cancer	NB4, Kas-1, MV4-11, and U937	0.5 to 1 μM	48 h	By concentrating on FTO/m6A and its ancillary pathways and ◙ AML leukemogenesis.	[79]
42.	Blood cancer	HL60	12.8–19.2 μM	48 h	▲GR mRNA expression, ◘ G0/G1- phase arrest, ◙ proliferation.	[80]
43.	Blood cancer	NB4	1.56, 3.12, and 6.25 μg/mL	5 days	▲Bactericidal activity, ◘ granulocyte differentiation via ▲PU.1, CEBPβ, and activating CBL-ERK1/2 pathway, ◙ proliferation.	[81]
44.	Blood cancer	THP-1	1.8, 3.0, and 4.3 μM	48 h	◙ Selectin-mediated cell adhesion.	[82]
45.	Brain cancer	U87	1–8 μM	48 h	▼ PI3K/Akt and ERK pathway, ♦ JNK, ◙ proliferation, Enhancement of apoptosis.	[83]
46.	Brain cancer	Primary microglia cells	0, 0.1, 0.25, 0.5, 1, 2, 4 μM	24 h	◘ Acute inflammatory depressive-like behaviors and microglia ♦, ◙ downstream TLR4/ NF-κB pathway.	[24]
47.	Brain cancer	C6	2.8–128 μM	4 days	◘ Differentiation, ◙ growth.	[84]
48.	Brain cancer	Shh Light II and DAOY	3 μM	36 h	◙ Cell proliferation, ▼ mRNA in Gli1 and Ptch1, GLI-luciferase activity and Hh signaling.	[85]
49.	Brain cancer	C6 rat glioma cells	1–20 μM [PGE2] 10–100 μM [Ca^2+^]	24 h	◙ PGE2 production, ◙ cyclooxygenase activity, and an elevation of [Ca^2+^].	[86]
50.	Brain cancer	PC12	0.125–2 μg/mL	24 h	Controls nuclear and mitochondrial GR translocation, partially reversing mitochondrial dysfunction, ◙ mitochondrial apoptotic pathway, ♦ GR-dependent survival pathway.	[87]
51.	Brain cancer	PC12	200, 300, and 400 μg/mL	6–96 h	▼PC12 cells’ apoptosis by reducing ROS and ◙ oxidative damage caused by MAPK.	[29]
52.	Ovarian cancer	SKOV3	2 μM	48 h	◘ Intracellular ROS accumulation, Enhancement of apoptosis, Chemosensitization (CDDP).	[51]
53.	Ovarian cancer	A2780s, A2780cp, Hey, SKOV3	1, 2 μM	24 h	▲ Ca^2+^ concentration, ◘ MMP loss, ♦ CaMKI, ◙ PPM1D, Promotion of mitochondrial fission, ◘ G2/M arrest. Chemosensitization (CDDP).	[88]
54.	Prostate cancer	DU145	2.5–50 μM	24 h	▲ p53, ◙ proliferation, ◘ G0/G1-phase arrest, ◘ of apoptosis.	[89]
55.	Prostate cancer	DU145, CWR22Rv1	5, 10 μM	24 h, 48 h, 72 h	◙ GSK3β/β-catenin pathway in CWR22Rv1, Suppression of proliferation, metastasis and invasion.	[90]
56.	Osteosarcoma	143B, MG-63	80 μM	24 h, 48 h, 72 h	♦ p53 pathway, ◘ apoptosis, ◘ G0/G1-phase arrest, ◙ proliferation.	[91]
57.	Osteosarcoma	U2	5–20 μM	24 h, 36 h, 48 h	◙ Akt and ERK pathway, ◙ proliferation, invasion, and migration, ◘ apoptosis.	[92]
58.	Colon cancer	SW480 and SW620	50 μg/mL	24 h	Promote apoptosis, ◙ PI3K/Akt/mTOR pathway and proliferation.	[93]
59.	Colon cancer	HT-29	10 μg/mL	24 h	Apoptosis of HT29 ▲, TRAIL, TRAIL-R and caspase10 and/or caspase8 ▼.	[94]
60.	Lymphoid tissue	Mouse T cells	5–15 μM	48 h	◙ T cell proliferation, ♦ NF-κB, NF-AT, and AP-1 signal pathways, ◙ cytokine secretion, IL-2 receptor expression.	[95]
61.	Lymphoid tissue	Rat basophilic leukemia-2H3 cells	50 μg/mL	1 h	◙ Intracellular calcium mobilization, ROS, cell degranulation, and tyrosine phosphorylation, ◙ gene ♦ of Cdc42 and c-Fos.	[96]
62.	Thyroid carcinoma	ARO, 8305C, SW1736	5–20 μM	12 h, 24 h, 48 h	♦ p53 pathway, ◙ proliferation, ◘ G1-phase arrest, ◘ apoptosis.	[97]
63.	Melanoma	A375.S2	5–20 μM	30 min	♦ JNK, p38, and p53, ◙ proliferation, ◘ apoptosis.	[98]
64.	Pancreatic cancer	BxPC3	1–8 μM	48 h and 72 h	♦ MKK4-JNK pathway, ◙ proliferation, ◘ apoptosis.	[99]
65.	Gastric cancer	SGC-7901, MGC-803, and HGC-27	2.5 μg/mL	72 h	◙ IKK β/NF-κB pathway, ◘ both cell autophagy and apoptosis.	[100]

▲—Increase, ▼—Decrease, ◙—Inhibition, ◘—Induces, ♦—Activation.

**Table 2:** Anticancer effects of SSD in vivo.

S. No.	Cancer Type	Model	Dose/Conc.	Exposure (Days)	Route of Administration	Effects on Signaling Pathways	Reference
1.	Lung cancer	HCC827/GR cells xenograft tumor in nude mice	5, 10 mg/kg	14 days	Intraperitoneal (IP)	◙ Growth, ◘ apoptosis, Chemosensitization (gefitinib)	[50]
2.	Lung cancer	BLM (5 mg/kg)-induced PF mice	2 mg/kg	28 days	IP	Alleviated pulmonary alveolitis, pulmonary fibrosis and cell apoptosis. ◙ Caspase-3, FN, Wnt and β-catenin, E-cad upregulated.	[53]
3.	Lung cancer	VILI rats			IP	▼ MIP-2, IL-6, and TNF-α and ▲ TGF-β1 and IL-10.	[101]
4.	Breast cancer	BALB/c mice (female, 4–6 weeks old)	1 or 5 mg/kg	3 days	Tail vein	◙ p-Akt and p-ERK.	[54]
5.	Breast cancer	MCF-7/ADR cells xenograft tumor in nude mice	5 mg/kg	20 days	IP	◙ Growth, ◙ P-gp expression, Reversal of MDR without toxic effect.	[58]
6.	Liver cancer	HSVtk/Hep3B cells xenograft tumor in nude mice	10 mg/kg	33 days	IP	◙ Growth, ◘ apoptosis, Chemosensitization (HSVtk/GCV)	[68]
7.	Liver cancer	Eight-week-old male C57BL/6J mice.	5, 10, and 20 mg/kg	4 weeks	Intragastric (IG)	◙ FA synthesis by retaining SREBP1c, ♦ INSIG1, INSIG2, and PPARα. ◘ FA catabolism in WAT.	[64]
8.	Liver cancer	BALB/c nude mice bearing SMMC-7721 xenograft tumor,	0.75 mg/kg	Thrice a week for 2 weeks	IP	◙ HIF-1α	[102]
9.	Liver cancer	Fish–hybrid grouper	100, 200, 400, and 800 mg/kg	56 days	IP	▼ IL-6, TNF-α, and IL-1β levels in liver tissue, and markedly immune inflammatory response and ◙ apoptosis.	[65]
10.	Liver cancer	DEN-induced Sprague Dawley rat HCC model	2 mg/kg	17 weeks	IP	◙ C/EBPβ and COX-2	[103]
11.	Liver cancer	C57/BL6rats	2 mg/kg 0.3 and 0.6 μg/mL	5 days	IP	◙ NF-κB and STAT3-mediated inflammatory signal pathway.	[44]
12.	Liver cancer	Hepatic fibrosis rats	1.0, 1.5, and 2.0 mg/kg	6 weeks	IP	◙ Liver TNF- α, IL-6, and NF-κB p65 expression and ▲ I-κBα activity.	[104]
13.	Liver cancer	SD rats	0.03% SSD	16 weeks	IG	▼Syndecan-2, MMP-2, MMP-13, and TIMP-2 tissue.	[33]
14.	Liver cancer	SD rats	0.75, 1.50 mg/kg			▼Serum corticosterone levels, BDNF, neurons generations, GR expression, and nuclear translocation▲.	[105]
15.	Liver cancer	SD male rats	1.0 mg/kg	Once a day for 18 days	IP	◙ Angiogenesis of DEN-induced hepatocarcinogenesis, ◙ Ang-2, and VEGF.	[106]
16.	Kidney cancer	Male C57BL/6J mice	10 mg/kg	3 days	IP	▼ Kidney injury and inflammation, ◘ SIRT1, ◙ IL-1B, NLRP3, SIRT1, and ROS.	[73]
17.	Kidney cancer	Wistar rats–Anti-Thy1 mAb 1–22–3-induced rat model of glomerulonephritis	0.6 or 1.8 mg/kg	31 days	IP	▼ TGF-β1 and type I collagen.	[107]
18.	Kidney cancer	LPS-induced mice	5, 20 mg/kg	1 week	IG	◙ FOSL1, TCF7, ◙ MMP9 expression and ▼ renal inflammation and ◘ cell apoptosis.	[108]
19.	Blood cancer	C57BL6/J mice	6, 12 mg/kg	6 days	IP	▲ Bactericidal activity, ◘ granulocytic differentiation by ♦ CBL-ERK1/2 pathway.	[81]
20.	Anti-Tumor activity	A549 cells-bearing nude mice	1.0 mg/kg		IG	◘ Apoptosis, ◙ COX-2.	[109]
21.	Brain cancer	MB allo-graft mice	10 mg/kg	18 days	IP	◙ Tumor growth, ▼Gli1, mRNA, ◙ Hh signaling pathway by targeting SMO.	[85]
22.	Brain cancer	Male ICR mice (18–22 g; 6–8 weeks old)	1 mg/kg	7 days	IG	▲LPS-induced inflammation, ◙ LPS-induced microglia activation and neuroinflammation. ▼HMGB1/TLR4/NF-κB.	[24]
23.	Brain cancer	3xTg mice (age, 9 months; weight, 30–35 g)	10 mg/kg	28 days	Oral	▼Cell apoptosis and inflammation, ◙ NF-κB activation. ◙ activation of microglia and astrocytes.	[110]
24.	Thyroid cancer	ARO cells xenograft tumor in nude mice	5–20 mg/kg	4 weeks	Oral	◙ Tumor growth.	[97]

▲—Increase, ▼—Decrease, ◙—Inhibition, ◘—Induces, ♦—Activation.

## Data Availability

Not applicable.
